# Postural Communication of Emotion: Perception of Distinct Poses of Five Discrete Emotions

**DOI:** 10.3389/fpsyg.2017.00710

**Published:** 2017-05-16

**Authors:** Lukas D. Lopez, Peter J. Reschke, Jennifer M. Knothe, Eric A. Walle

**Affiliations:** Psychological Sciences, University of California, Merced, MercedCA, USA

**Keywords:** emotion, emotion expression, emotion perception, postural communication, dimensional ratings

## Abstract

Emotion can be communicated through multiple distinct modalities. However, an often-ignored channel of communication is posture. Recent research indicates that bodily posture plays an important role in the perception of emotion. However, research examining postural communication of emotion is limited by the variety of validated emotion poses and unknown cohesion of categorical and dimensional ratings. The present study addressed these limitations. Specifically, we examined individuals’ (1) categorization of emotion postures depicting 5 discrete emotions (joy, sadness, fear, anger, and disgust), (2) categorization of different poses depicting the same discrete emotion, and (3) ratings of valence and arousal for each emotion pose. Findings revealed that participants successfully categorized each posture as the target emotion, including disgust. Moreover, participants accurately identified multiple distinct poses within each emotion category. In addition to the categorical responses, dimensional ratings of valence and arousal revealed interesting overlap and distinctions between and within emotion categories. These findings provide the first evidence of an identifiable posture for disgust and instantiate the principle of equifinality of emotional communication through the inclusion of distinct poses within emotion categories. Additionally, the dimensional ratings corroborated the categorical data and provide further granularity for future researchers to consider in examining how distinct emotion poses are perceived.

## Introduction

Emotional expressions communicate individuals’ mental states, goals, and likely behaviors (see Fridlund, 1994; Parkinson, 2005). The study of emotion expressions has traditionally focused on the face as the primary channel through which emotion is communicated and appreciated (e.g., Ekman and Friesen, 1971, 1978; Öhman and Dimberg, 1978; Russell and Bullock, 1985; Russell, 1994; Ekman, 1999). However, a growing body of research demonstrates that other expressive channels are also important for emotion perception (for reviews, see Matsumoto et al., 2010; Barrett et al., 2011; de Gelder and Van den Stock, 2011a; Hassin et al., 2013). Non-facial signals of emotion include the voice (Banse and Scherer, 1996; Vaish and Striano, 2004), body posture (Coulson, 2004; Kleinsmith and Bianchi-Berthouze, 2007; Aviezer et al., 2008a,b, 2011; de Gelder and Van den Stock, 2011b), movement (De Meijer, 1989; Atkinson et al., 2007), and the social context (Masuda et al., 2008; Mumenthaler and Sander, 2012).

Of the above modalities, the role of posture in the perception of emotion has been of increased empirical interest in the field of emotion (see Aviezer et al., 2008a; Barrett et al., 2011; Hassin et al., 2013). However, fundamental questions relating to the perception of emotion postures remain. Specifically, prior research has not determined (1) whether a posture of disgust can be reliably identified, (2) how different postural poses within emotion categories are recognized, particularly those expressing distinct action tendencies, and (3) the correspondence between categorical and dimensional ratings of emotion postures. This investigation addressed each of these gaps in the literature.

### Postural Display of Emotion

The role of bodily posture is centrally featured in classic writings of emotion (see Darwin, 1872; James, 1884; Dewey, 1894). Bodily posture conveys action tendencies associated with corresponding emotions (Frijda, 1986). For example, a fear posture typically involves an avoidant and protective physical response to an external referent, whereas an anger posture is associated with the individual extending toward a referent and becoming larger. More recent empirical research demonstrates that even relatively small differences in body posture and movement can influence emotion perception and categorization (Dael et al., 2012).

Of particular interest is work investigating the effect of body posture on the perception of affective expressions (Meeren et al., 2005; Van den Stock et al., 2007; Aviezer et al., 2008b, 2011; de Gelder, 2009). For example, a disgust face transposed onto a posture demonstrating a punching motion is perceived as a facial expression of anger (Aviezer et al., 2008b). Such research makes central the need to more carefully consider the perception of emotion as communicated through bodily posture. However, although recent studies have manipulated the postures on which facial expressions were placed (e.g., Aviezer et al., 2008b), these postures often included additional non-postural elements (e.g., a headstone in the background; a soiled undergarment) that may have influenced emotion perception independently of the accompanying posture. Moreover, studies specifically examining the perception of emotion postures frequently display the body in conjunction with corresponding facial expressions (e.g., Bänziger et al., 2012), creating an obvious confound with regards to the perception of the posture in and of itself. More careful study of postural communication of emotion is critical given the influential role of bodily posture in emotion perception.

#### Limitations of Prior Emotion Posture Research

Though informative, prior research examining postural displays of emotion has suffered from three crucial limitations.

First, to our knowledge, it has yet to be established whether disgust is reliably recognized when communicated through posture alone. Previous research examining static postural displays of emotion have either neglected to include a disgust posture (e.g., de Gelder and Van den Stock, 2011b) or failed to identify a reliably recognizable posture of disgust (e.g., Atkinson et al., 2004; Coulson, 2004). Furthermore, as noted above, studies including a static disgust posture often include contextual information in the image (e.g., Aviezer et al., 2008b). The lack of an identifiable disgust posture is problematic given its categorization as a basic emotion (see Ekman, 1992) and unique adaptive function for identifying and avoiding potentially dangerous or offensive substances (see Darwin, 1872; Saarni et al., 2006). Empirical confirmation of a static disgust posture, void of other emotionally relevant elements, is needed.

Second, studies examining the perception of emotion postures typically include only a single pose for each emotion (Atkinson et al., 2004; de Gelder and Van den Stock, 2011b), thereby ignoring the equifinality of emotional expression – that a discrete emotion can be made manifest in different ways (see Campos et al., 2004). For example, anger postures typically feature an individual raising a fist as if to strike out at a potential threat. However, one can be angry without displaying other-directed actions, such as displaying self-directed behavior (e.g., “ripping one’s hair out”; see Frijda, 2007). Specific to posture, Frijda describes a number of distinct action tendencies corresponding with particular emotions. Importantly, action tendencies are not necessarily unique to a single emotion (Frijda, 1986), but may be shared by different emotions, and even vary *within* emotions (Frijda, 2007). Work in affective neuroscience indicates that postural and facial expressions of emotion are categorized based on their affective meaning, not simply their physical features (Tamietto et al., 2009; Diano et al., 2017), and that these processes operate at both the conscious and automatic level (see Filmer and Monsell, 2013; Celeghin et al., 2015), suggesting that discrete emotions can be communicated through distinct forms of expression. Furthermore, the equifinality of emotional expression is a fundamental principle of affective communication (Campos et al., 2004), but is lacking in studies of emotional postures. Examining the categorization of distinct poses within emotion categories is needed to explore this theoretical principle.

Finally, previous studies of emotion posture have assessed categorization of discrete emotions *or* ratings along dimensions of valence and arousal, but not *both*. Inclusion of both categorical and dimensional ratings is necessary to examine the coherence (or lack thereof) between discrete and dimensional accounts of emotion in postural communication. Prior research on emotion perception has noted coherence in categorical and dimensional ratings of both facial and vocal expressions of emotion (e.g., Laukka et al., 2005; Mendolia, 2007; Fujimura et al., 2012). Evidence supporting such coherence in the perception of emotion postures remains unknown. Separate lines of research have examined the perception of emotion postures using affective dimensions (e.g., Kleinsmith and Bianchi-Berthouze, 2007) or categorization (e.g., de Gelder and Van den Stock, 2011b). However, to our knowledge no previous study has included both discrete categorization *and* ratings of valence and arousal of emotion postures.

### The Present Study

This study examined adult ratings of postures expressing five discrete emotions: joy, sadness, fear, anger, and disgust. The study was guided by three primary aims to address the above limitations. First, we attempted to validate a static postural display of disgust. Second, we examined participant emotion categorization of distinct bodily postures conveying different action tendencies (e.g., anger expressed as striking out vs. reaching upward in exasperation). Finally, we assessed participant ratings of valence and arousal for each posture to corroborate categorical and dimensional properties of postural communications of emotion.

## Materials and Methods

### Stimuli Construction

Two actors (one male, one female) posed postural expressions of five discrete emotions: joy, sadness, fear, anger, and disgust. Actors wore gender-neutral clothing (collared shirt or sweater with jeans) that covered their arms and legs. Pictures were taken with an 8 megapixels digital camera in front of a white background under controlled lighting. Images were digitally altered using Adobe Photoshop to remove the head and neck of each actor, resulting in each image displaying the body from the collar down. Additionally, all images were converted to gray scale to minimize any effect of clothing color and actor skin tone (visible on the hands) on participants’ ratings.

**FIGURE 1:**
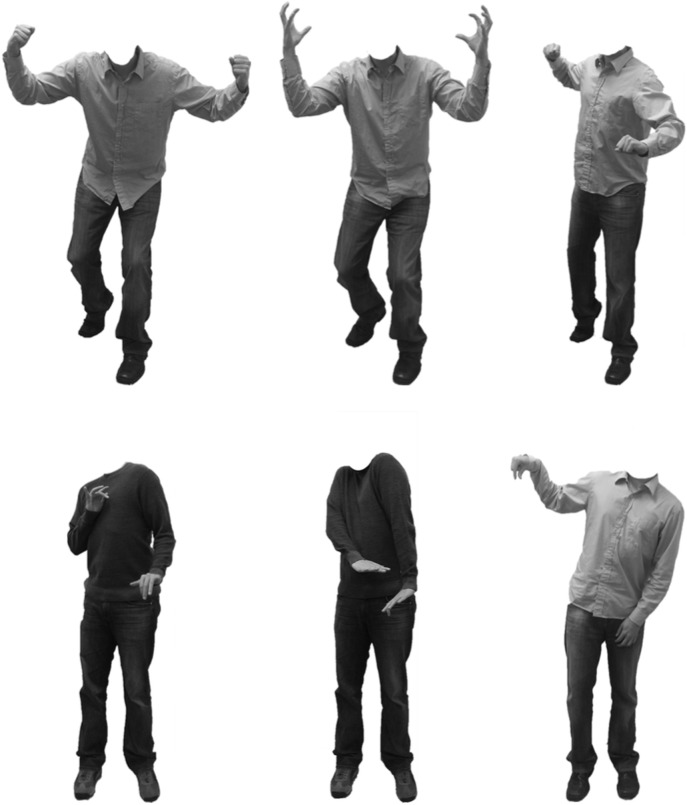
**Examples of the male actor displaying distinct poses of anger (**Top**, L–R: Anger 1, Anger 2, Anger 3) and disgust (**Bottom**, L–R: Disgust 1, Disgust 2, Disgust 3)**.

Each discrete emotion category included a bodily pose similar to previously validated stimuli (Aviezer et al., 2011; de Gelder and Van den Stock, 2011b), as well as iterations of related, but structurally novel, postural expressions. The resulting set of 30 stimuli consisted of distinct poses for joy (3), sadness (4), fear (2), anger (3), and disgust (3), each including a male and female version (see **Figure 1**, for examples^1^). The number of poses per emotion varied as a function of the flexibility with which particular emotions are likely to be expressed. For example, it is theorized that there are many ways to express sadness and anger, but likely fewer ways with which to express fear (see Frijda, 1986). All stimuli subtended a vertical visual angle of 11.7° and an average horizontal visual angle of the torso of 2.27° (*SD* = 0.15°) when viewed from a distance of 60 cm, and exuded an average luminance of 63.78 cd/m^2^ (*SD* = 19.21). Stimuli did not differ significantly on these low-level features across emotions [Subtension: *F*(4,15) = 2.29, *p* = 0.11; Luminance: *F*(4,15) = 1.55, *p* = 0.24] or within emotions [Subtension: *F*(7,15) = 1.16, *p* = 0.38; Luminance: *F*(7,15) = 0.28, *p* = 0.95].

### Procedures

Separate samples were recruited to complete the categorization and dimensional portions of the study to prevent potential carryover effects across ratings. The UC Merced Institutional Review Board approved all procedures and all participants provided written informed consent.

#### Discrete Emotion Categorization

Twenty-six undergraduate students (18 female; mean age = 21.15, *SD* = 4.76; 13 Hispanic, 6 Asian American, 4 European American, 1 African American, and 2 of mixed ethnicity) from the University of California, Merced, participated in the categorization portion of the study in exchange for course credit. A power analysis based on findings from a similar study (de Gelder and Van den Stock, 2011b) indicated that this sample size was sufficient to provide adequate power for detecting differences in emotion categorization. The images were presented in a randomized order using Qualtrics survey software on individual computers in a campus computer lab. Three graduate student researchers monitored all participants during completion of the study. Each postural stimulus was presented with the following prompt: “Select the emotion that best describes the way the person is feeling”. Below each prompt the participant was required to choose from a list of five discrete emotions listed vertically in a set order: joy, sadness, fear, anger, disgust. The image remained on the screen until the participant selected an emotion from the list. Participants identified all 30 images in a single sitting lasting approximately 10 min.

#### Dimensional Ratings

Twenty-one undergraduate students (12 female; mean age = 19.04, *SD* = 1.28; 13 Hispanic, 2 Asian American, 1 European American, 2 African American, 1 Pacific Islander, and 2 of mixed ethnicity) from the University of California, Merced, participated in the dimensional rating portion of the study in exchange for course credit. This sample size was similar to that from previous research examining ratings of affective dimensions (Russell et al., 1989). The setting was identical to the emotion categorization procedure, and all instructions and procedures were administered using Qualtrics survey software. Participants were first provided with a description of the 9 × 9 Affect Grid used to rate the images (see Russell et al., 1989). Participants were instructed that the *x*-axis of the grid represented valence (extreme positive valence on the far right and extreme negative valence on the far left) and the *y*-axis represented arousal (excitement on the uppermost section and sleepiness on the bottommost section). Participants viewed each image and were asked, “Using the affect grid, indicate how this person feels”. The postural stimuli were presented individually in a randomized order above the affective grid. Labels for the anchors of each dimension were included as reminders for each dimension (valence: positive vs. negative; arousal: excitement vs. sleepiness). The image remained on the screen until the participant selected a coordinate on the affective grid. Participants rated all 30 images in a single sitting lasting approximately 10 min. Valence and arousal ratings falling more than 2 *SD*s above or below the mean for each posture version were determined to be outliers, resulting in the exclusion of 49 of the 630 total ratings.

## Results

The data analysis proceeded in two phases. First, we examined categorical agreement and systematic miscategorization for each posture, and whether particular poses were more recognizable than others in the same emotion category. Second, we explored participant ratings of valence and arousal for each discrete emotion, and whether particular poses within emotion categories varied along each dimension.

Preliminary analyses revealed that the effects of image gender, visual angle, and luminance were not related with participants’ categorizations of the target emotion and ratings of valence and arousal. Thus, these variables were excluded from subsequent analyses.

### Emotion Categorization

The overall percent agreement for the posture images was high (79%) and the corresponding Cohen’s kappa value indicated substantial inter-rater agreement (κ = 0.64; Landis and Koch, 1977). The majority of images were categorized as the target emotion significantly above chance levels (one-tailed binomial tests, *p*s < 0.003), with the exception of the female Joy 3 posture (*p* = 0.23) and the male Sadness 4 posture (*p* = 0.06).

Bonferroni-corrected paired *t*-tests (α = 0.0125) revealed that 14 of the 15 posture versions were identified as the target emotion significantly more frequently than any other emotion category (all *p*s < 0.003). The exception was the Joy 3 posture, which was rated as joy in the majority of instances (*M* = 52%), but not significantly more than it was miscategorized as anger, (*M* = 33%), *t*(25) = 1.31, *p* = 0.20, CI [-0.20, 0.59]. The confusion matrix presented in **Table 1** provides the proportions of participant emotion classifications for the emotion postures.

**Table 1:** Proportion of emotion categorizations and mean dimensional ratings for posture versions.

	Categorizations					Dimensions	
		
Posture	Joy	Sadness	Fear	Anger	Disgust	Valence	Arousal
Joy overall	**0.69**	0.02	0.03	0.24	0.03	5.36 (1.50)	6.16 (1.16)
Joy 1	**0.81**	0.02	0.02	0.15	0.00	5.63 (1.40)	5.93 (1.24)
Joy 2	**0.73**	0.00	0.04	0.23	0.00	5.74 (1.66)	6.52 (1.17)
Joy 3	**0.52**	0.04	0.02	0.33	0.10	4.74 (1.27)	6.02 (1.03)
Sadness overall	0.11	**0.72**	0.13	0.02	0.02	3.45 (1.52)	4.37 (1.61)
Sadness 1	0.02	**0.87**	0.10	0.00	0.02	3.38 (1.04)	3.38 (1.17)
Sadness 2	0.12	**0.65**	0.23	0.00	0.00	2.74 (1.14)	3.95 (1.41)
Sadness 3	0.17	**0.75**	0.06	0.00	0.02	3.83 (2.23)	5.14 (2.09)
Sadness 4	0.12	**0.62**	0.13	0.10	0.04	3.89 (1.15)	5.05 (0.72)
Fear overall	0.01	0.00	**0.88**	0.01	0.10	2.14 (0.95)	7.36 (1.26)
Fear 1	0.02	0.00	**0.92**	0.00	0.06	2.05 (1.16)	7.57 (1.27)
Fear 2	0.00	0.00	**0.85**	0.02	0.13	2.24 (0.70)	7.14 (1.25)
Anger overall	0.07	0.00	0.02	**0.90**	0.01	2.47 (2.04)	8.04 (1.16)
Anger 1	0.17	0.00	0.04	**0.79**	0.00	3.98 (2.58)	7.79 (1.31)
Anger 2	0.00	0.00	0.00	**0.98**	0.02	1.62 (1.00)	8.02 (1.32)
Anger 3	0.04	0.00	0.02	**0.94**	0.00	1.74 (1.13)	8.34 (0.67)
Disgust overall	0.08	0.01	0.15	0.01	**0.76**	3.84 (1.78)	5.96 (1.29)
Disgust 1	0.06	0.00	0.15	0.02	**0.77**	3.36 (1.27)	6.26 (1.23)
Disgust 2	0.02	0.00	0.25	0.00	**0.73**	3.05 (1.42)	5.88 (1.47)
Disgust 3	0.15	0.02	0.04	0.00	**0.79**	5.12 (1.88)	5.74 (1.16)


*Target emotion is in bold. *SD*s included in parentheses for ratings of valence and arousal. See Supplementary Table 1 for data of each image separated by gender.*

#### Posture Miscategorizations

Bonferroni-corrected paired *t*-tests (α = 0.0083) were conducted to compare proportions of non-target emotion ratings to examine possible systematic miscategorizations of each posture pose.

##### Joy

No significant miscategorizations between non-target emotion ratings were found for Joy 1 (*p*s > 0.018). However, Joy 2 and Joy 3 were both falsely identified as anger significantly more often than sadness, *t*(25) = 3.64, *p* = 0.001, CI [0.05, 0.41], *t*(25) = 3.64, *p* = 0.001, CI [0.06, 0.52], respectively, and fear *t*(25) = 3.08, *p* = 0.005, CI [0.05, 0.41], *t*(25) = 4.17, *p* < 0.001, CI [0.10, 0.52], respectively. Additionally, Joy 2 was miscategorized as anger significantly more often than disgust, *t*(25) = 3.64, *p* = 0.001, CI [0.05, 0.41].

##### Sadness

Miscategorizations of Sadness 1, Sadness 3, and Sadness 4 did not differ systematically (*p*s > 0.03). However, Sadness 2 was miscategorized as fear significantly more often than anger, *t*(25) = 3.64, *p* = 0.001, CI [0.05, 0.42], and disgust, *t*(25) = 3.64, *p* = 0.001, CI [0.05, 0.42].

##### Fear

Miscategorizations of Fear 1 did not vary systematically (*p*s > 0.09). However, Fear 2 was incorrectly identified as disgust significantly more often than joy, *t*(25) = 3.04, *p* = 0.006, CI [0.01, 0.26], and sadness, *t*(25) = 3.04, *p* = 0.006, CI [0.01, 0.26].

##### Anger

There were no significant miscategorizations for Anger 1, Anger 2, or Anger 3 (*p*s > 0.01).

##### Disgust

There were no significant miscategorizations for Disgust 1 or Disgust 3 (*p*s > 0.02). However, Disgust 2 was incorrectly categorized as fear significantly more than joy, *t*(25) = 2.90, *p* = 0.008, CI [0.00, 0.46], sadness, *t*(25) = 3.35, *p* = 0.003, CI [0.04, 0.46], and anger, *t*(25) = 3.35, *p* = 0.003, CI [0.04, 0.46].

#### Recognition of Poses within Emotion Categories

Each emotion category had at least two unique postural poses for which both male and female versions were validated at or above 65% (see **Table 1**). Bonferroni-corrected^2^ paired *t*-tests comparing the frequency of responses matching the target emotion examined differences in poses within each emotion category to determine whether some versions were better recognized than others.

##### Joy

The Joy 1 pose was correctly categorized as joy significantly more than the Joy 3 pose, *t*(25) = 2.67, *p* = 0.01, CI [0.01, 0.57]. Differences in joy categorizations between Joy 1 and Joy 2, and Joy 2 and Joy 3, were not significant (*p*s > 0.36).

##### Sadness

Correct categorization of Sadness 1 was significantly more frequent than Sadness 4, *t*(25) = 3.35, *p* = 0.003, CI [0.04, 0.46]. There were no significant differences in correctly categorizing the other sadness poses (*p*s > 0.01).

##### Fear

There was no significant difference in fear categorizations between posture versions of fear (*p* = 0.21).

##### Anger

The Anger 2 pose was categorized as anger significantly more often than the Anger 1 pose, *t*(25) = 2.81, *p* = 0.01, CI [0.02, 0.38]. There were no significant differences in anger categorizations between the remaining anger poses (*p*s > 0.04).

##### Disgust

No significant differences in disgust categorizations were present between distinct poses of disgust (*p*s > 0.54).

### Dimensional Ratings

**FIGURE 2:**
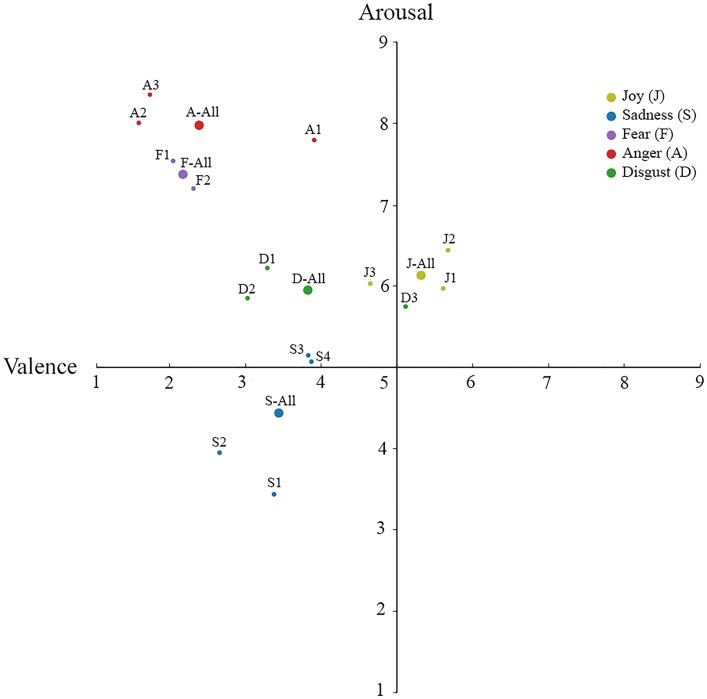
**Mean ratings of valence and arousal for each category of emotion.** The mean valence and arousal dimensions are plotted for each posture emotion category, as well as the unique poses within each emotion category.

Participant ratings of valence and arousal were analyzed using Bonferroni-corrected paired *t*-tests to examine differences in each dimension between emotion categories and between poses within the same emotion category. The mean ratings of valence and arousal dimensions for each pose are presented in **Table 1**. Additionally, the spatial coordinates of the valence and arousal ratings of each posture are plotted in **Figure 2**.

#### Dimensional Ratings between Emotion Categories

Ratings of posture valence differed significantly across most discrete emotions (α = 0.005; all *p*s < 0.001), with the exceptions of fear and anger (*p* = 0.32), and sadness and disgust (*p* = 0.14). Arousal ratings also demonstrated differentiation across most discrete emotions (all *p*s < 0.001), with the exception of the joy and disgust postures (*p* = 0.34), and fear and anger postures (*p* = 0.006).

#### Dimensional Ratings within Emotion Categories

##### Joy

No significant differences were found in the ratings of valence or arousal between the joy poses (all *p*s > 0.02).

##### Sadness

Analyses of the valence ratings for the sadness postures revealed that Sadness 2 was rated significantly more negative than Sadness 1, *t*(20) = 3.01, *p* = 0.006, CI [0.04, 1.25], and Sadness 4, *t*(18) = 2.99, *p* = 0.008, CI [0.01, 2.15]. With regards to arousal, Sadness 3 was rated significantly higher in arousal than Sadness 1, *t*(20) = 4.95, *p* < 0.001, CI [0.72, 2.80]. Additionally, Sadness 4 was rated significantly higher in arousal than Sadness 1, *t*(18) = 7.44, *p* < 0.001, CI [1.06, 2.46], and Sadness 2, *t*(18) = 4.24, *p* < 0.001, CI [0.34, 1.92]. No other differences in valence or arousal of sadness poses were statistically significant (all *p*s > 0.02).

##### Fear

Fear 1 and Fear 2 did not differ significantly in valence (*p* = 0.52). However, Fear 1 was rated significantly higher in arousal than Fear 2, *t*(20) = 2.15, *p* = 0.04, CI [0.01, 0.84].

##### Anger

The valence of Anger 1 was rated as significantly less negative than Anger 2, *t*(20) = 4.49, *p* < 0.001, CI [0.99, 3.73], and Anger 3, *t*(18) = 4.91, *p* < 0.001, CI [1.18, 3.92]. However, Anger 2 and Anger 3 did not differ significantly by valence (*p* = 0.87). Additionally, there were no significant differences in arousal between any anger poses (*p*s > 0.24).

##### Disgust

Analyses of disgust postures revealed that Disgust 3 was rated significantly less negative in valence than Disgust 1, *t*(20) = 4.12, *p* = 0.001, CI [0.64, 4.12], and Disgust 2, *t*(20) = 5.09, *p* < 0.001, CI [1.01, 3.14]. Valence ratings of Disgust 1 and Disgust 2 were not statistically different (*p* = 0.35). Additionally, no significant differences in ratings of arousal were present for any of the disgust poses (*p*s > 0.11).

## Discussion

Our examination of individuals’ perception of emotion postures had had three specific aims. First, we assessed participant categorization of images depicting five discrete emotion postures, including a disgust posture. Second, we examined whether the same emotion could be expressed using a variety of distinct poses. Third, postures were rated along the dimensions of valence and arousal to further assess the perceptual aspects of postural expressions of emotion. Below we discuss the findings relating to these aims and place the contribution of each in the context of prior research.

### Categorization of Discrete Emotion Postures

Participants accurately categorized postural expressions of five discrete emotions (i.e., joy, sadness, fear, anger, and disgust). The identification of a postural expression of disgust represents a novel contribution to the emotion literature, and to our knowledge is the first study to validate a static disgust posture in which no facial or contextual cues were present. Interestingly, disgust postures were identified at comparable, if not better, rates as other emotion postures.

Further examination of participant miscategorizations of emotion postures indicated that joy postures were most commonly miscategorized as anger, and sadness postures were sometimes confused with fear, particularly when the body was angled to one side (i.e., Sadness 2). The observed miscategorizations provide unique insight into the potential confusability of discrete bodily postures akin to structural similarities documented in facial expressions of emotion (Susskind et al., 2007). While such a bottom-up processing of emotional postures is possible, equally plausible is a top-down explanation centering on likely actions associated with the posture. For example, when disgust was miscategorized it was typically labeled as fear, specifically when the arms were outstretched in a downward motion (i.e., Disgust 2). This pattern of miscategorization may be due to the functionally similar action tendencies of these emotions to avoid a stimulus or source of threat (Fridja et al., 1989).

### One Emotion, Many Postures

Postures for each emotion were successfully categorized using multiple physically distinct poses. Notable exemplars included poses varying in the communicated action tendency, such as expressing “anger in” and “anger out” (see Frijda, 2007). While prior studies have included multiple actors expressing the same emotion (e.g., de Gelder and Van den Stock, 2011b), the pose and action tendency expressed for each emotion were typically invariant in such studies. Furthermore, although prior studies including multiple actors could be said to have inadvertently included distinctions in poses across actors, lack of statistical comparisons of such differences prevent any definitive conclusions on this point. Also, while the present study included a range of poses within each emotion category, there are likely numerous other poses of these emotions that are distinct from those tested. Emotion poses in the present study that resembled postures used in prior research (i.e., Joy 1, Fear 2, and Anger 3) were rated with similar or better accuracy than reported previously (Atkinson et al., 2004; de Gelder and Van den Stock, 2011b). It is also worth noting that although all postures in the current study were correctly categorized as the target emotion, some poses within emotion categories were more accurately recognized than others. For example, the anger pose featuring hands clawing upward in the air (Anger 2) was more accurately identified than the anger pose with clenched fists in the air (Anger 1). Interestingly, the latter was often confused as joy, which may be due to its perceptual similarity with bodily expressions of pride (e.g., Aviezer et al., 2008b; Tracy and Matsumoto, 2008).

Participants’ successful identification of perceptually distinct, yet categorically similar, emotion postures also substantiates a broader theoretical point: the equifinality of emotional expression. Researchers seeking a singular archetype expression of discrete emotions risk ignoring the underlying behavioral functions of different emotional responses (Campos et al., 1989). The present findings support the view that different action tendencies can be associated with the same emotion (Frijda, 2007). Thus, rather than utilizing a 1:1 mapping of expression and emotion, the processing of emotional communication likely relies on an appreciation for the nature of a social partner’s relation with the environment (see Barrett and Campos, 1987).

Furthermore, the inclusion of a variety of postural expressions of the same discrete emotion expands the repertoire of postures available to researchers and increases flexibility for conducting research on emotion perception. This is likely of particular relevance for studies integrating emotion postures with other emotional stimuli, such as faces or contextual scenes (e.g., Meeren et al., 2005; Aviezer et al., 2008b; Righart and de Gelder, 2008; Kret and de Gelder, 2010; Mondloch, 2012). For example, an anger posture depicting a raised fist toward a rival may be more contextually appropriate than the same posture directed toward a broken computer, for which the upward hands in exasperation is likely more ecologically valid. The present set of stimuli provides additional avenues for combining emotion postures with other emotion-related elements.

### Differences in Valence and Arousal of Emotion Postures

In addition to categorizing each postural pose of emotion, a separate group of participants rated the stimuli on dimensions of valence and arousal using the Affect Grid (Russell et al., 1989). The majority of postural expressions of discrete emotion categories were distinct in valence and arousal, though there were some exceptions. Fear and anger postures were similarly negative in valence and high in arousal despite typically being associated with opposing action tendencies. Additionally, valence ratings revealed that sadness and disgust postures were similarly negative, and that ratings of arousal did not differ for joy and disgust postures.

In general, the observed pattern of results is in line with previous research examining dimensional ratings of emotion facial expressions. However, it is notable that joy postures were rated less positively in valence than facial expressions of joy included in prior research (Russell and Bullock, 1985). This may help account for joy postures being consistently more difficult to validate in comparison with other bodily expressions of emotion (de Gelder and Van den Stock, 2011b). It is also interesting that valence and arousal varied between some poses belonging to the same emotion category. For example, Anger 2 (punching anger) and Anger 3 (inner anger) were rated as significantly more negative in valence than Anger 1 (both fists in the air). As stated earlier, these differences may suggest that some raters may have perceived Anger 1 as communicating pride, resulting in Anger 1 being rated less negatively valenced.

More generally, the present findings demonstrate the value of including both categorical and dimensional data to better understand differences in the perception of emotional communication. The dimensional data can help inform the selection of specific emotion postures for use with contextual cues. For example, high-arousal postures may exert increased influence on the perception of other modalities of emotional communication, such as the face (see Aviezer et al., 2012). Additionally, ratings of valence and arousal can allow investigators to compare emotion postures beyond their perceptual features (e.g., arms being up or arms being down) and consider similarities and distinctions in their communication of these dimensions.

### Additional Considerations

Several considerations relating to the limitations of the present study and opportunities for further research warrant mentioning. First, the procedures and design of the study may have limited participants’ responding. Our procedure utilized a forced-choice method using five basic emotion categories. Although this methodology is consistent with previous posture studies (e.g., Atkinson et al., 2004; de Gelder and Van den Stock, 2011b), a less constrained response set (e.g., including an ‘other’ or ‘fill-in-the-blank’ option) may have yielded greater variation in participant ratings. Thus, while our inclusion of five distinct responses may have been more liberal than other emotion classification studies (e.g., de Gelder and Van den Stock, 2011b), this design may have restricted the richness of participant differences in perceiving the emotion poses. This concern may be particularly relevant for the parsing of emotions within an emotion category (e.g., ‘solemn’ vs. ‘sad’ vs. ‘depressed’) or identifying other discrete emotion categories (e.g., pride, shame, awe). The absence of a debriefing session limits our knowledge of how appropriately the provided emotion labels accurately captured participant perceptions of the poses. Additionally, the lack of a neutral posture precludes the ability to compare the present postures with a “control” posture (though whether a neutral expression would provide such a control is debatable; see Donegan et al., 2003; Somerville et al., 2004). Although, previous studies have similarly omitted inclusion of a neutral posture or a neutral option in categorization procedures (Atkinson et al., 2004; de Gelder and Van den Stock, 2011b), the inclusion of such a posture or option would have provided greater distinction in assessing the categorical and dimensional ratings for the emotions of interest.

Second, it is important to consider aspects relating to the construction of the stimuli. The decision to exclude the head of the actor in our posture images may have eliminated a relevant element for emotion perception (Atkinson et al., 2004; de Gelder and Van den Stock, 2011b). Inclusion of the head with a blurred face may have increased the validation scores in the present study. For example, including the head being pulled back so as to avoid sensory contamination could further increase categorization for disgust poses (Rozin and Fallon, 1987). While the positioning of the head can certainly provide information regarding the communicated emotion (Dael et al., 2012), the decision to omit the head from the present set of stimuli was made to accentuate the role of the body. Importantly, it seems unlikely that our decision to exclude the head from our images artificially inflated participants’ ratings – in fact, the obtained ratings may have been more robust had head orientation been included. Additionally, each of the included emotion poses was artificially staged. Research examining facial expressions of emotion has noted that classic displays (e.g., Ekman and Friesen, 1976) are often different from those deemed naturalistic (e.g., Gosselin et al., 1995; Carroll and Russell, 1997; Scherer and Ellgring, 2007) or observed spontaneously (for reviews, see Fernández-Dols and Crivelli, 2013; Reisenzein et al., 2013). Whether such discrepancies exist in postural expressions of emotion remains to be studied. Comparison of the present stimuli with those naturally observed would help to account for any perceived artificiality of the stimuli.

Finally, although the present study isolated postural communication of emotion, it is crucial that research on emotion perception also consider the gestalt of emotion contexts. We encourage future studies to explore how emotion perception is affected by different elements of relational contexts, and particularly how such elements may interact with one another. For example, a disgust face superimposed onto an angry posture with both fists raised (i.e., Anger 1 pose) in the context of a victorious sporting event may be judged as pride despite neither the face nor the posture being categorized as pride when viewed in isolation (Aviezer et al., 2012). Examining how specific emotion-related elements (e.g., face, posture, voice, physical environment, cultural context, personal history of the protagonist) differentially influence emotion perception is a topic on which researchers have only begun to scratch the surface (for an excellent example of such research, see Van den Stock et al., 2007).

## Conclusion

Ultimately, this study contributes to the literature by confirming the recognition of distinct postures for five discrete emotions, including disgust, highlights the importance of equifinality of emotional communication, and builds on research examining the coherence of categorical and dimensional ratings of emotion. Moreover, the set of emotion posture images created and validated in this study can be used in future emotion perception research. We urge that such research examine how emotion postures are perceived when combined with other emotionally relevant information.

## Ethics Statement

This study was carried out in accordance with the recommendations of the Human Subjects Protection –Institutional Review Board at the University of California, Merced. All subjects gave written informed consent. The protocol was approved by the UC Merced Institutional Review Board.

## Author Contributions

All authors have contributed equally in study design, data collection, data analysis, and manuscript preparation.

## Conflict of Interest Statement

The authors declare that the research was conducted in the absence of any commercial or financial relationships that could be construed as a potential conflict of interest.

## Footnotes

1 The full set of pictures can be obtained by contacting the corresponding author or visiting [http://idlab.ucmerced.edu]. See Supplementary Figure 1 for low-resolution images of each picture.

2 Adjusted *p*-values for categorical and dimensional comparisons within emotion categories were: Joy α = 0.0167; Sadness α = 0.0083; Fear α = 0.05; Anger α = 0.0167; Disgust α = 0.0167.
