# Supplementary material for: Postural Communication of Emotion: Perception of Distinct Poses of Five Discrete Emotions
Source: Front Psychol. 2017 May 16;8:710. doi: 10.3389/fpsyg.2017.00710 (PMC5432628; doi:10.3389/fpsyg.2017.00710)
Supplement: Supplementary file 1 [file Table_1.pdf]

Supplementary Table 1.

Proportion of emotion categorizations and mean dimensional ratings for each posture.

		Categorizations					Dimensions	
Posture	Gender	Joy	Sadness	Fear	Anger	Disgust	Valence	Arousal
Joy 1	M	**0.88**	0.00	0.04	0.08	0.00	5.57 (1.89)	5.52 (1.72)
	F	**0.77**	0.00	0.00	0.23	0.00	5.43 (1.63)	5.86 (1.59)
Joy 2	M	**0.81**	0.00	0.04	0.15	0.00	6.43 (1.69)	6.62 (1.20)
	F	**0.65**	0.00	0.04	0.31	0.00	5.00 (2.07)	6.14 (1.93)
Joy 3	M	**0.58**	0.00	0.04	0.31	0.08	4.62 (1.50)	6.14 (1.24)
	F	**0.46**	0.08	0.00	0.35	0.12	4.95 (1.91)	5.90 (1.30)
Sadness 1	M	0.04	**0.81**	0.12	0.00	0.04	3.67 (1.28)	3.38 (1.69)
	F	0.00	**0.88**	0.12	0.00	0.00	3.05 (1.32)	3.71 (1.55)
Sadness 2	M	0.08	**0.70**	0.23	0.00	0.00	2.95 (1.66)	3.90 (1.64)
	F	0.12	**0.73**	0.12	0.00	0.04	2.86 (1.65)	4.05 (1.63)
Sadness 3	M	0.04	**0.85**	0.12	0.00	0.00	3.19 (2.20)	5.10 (2.32)
	F	0.19	**0.65**	0.12	0.00	0.04	4.48 (2.99)	5.19 (2.25)
Sadness 4	M	0.12	**0.50**	0.19	0.12	0.08	3.86 (1.42)	5.00 (1.34)
	F	0.12	**0.73**	0.12	0.00	0.04	3.62 (1.43)	5.19 (1.44)
Fear 1	M	0.00	0.00	**1.00**	0.00	0.00	1.90 (1.26)	8.10 (1.14)
	F	0.04	0.00	**0.85**	0.00	0.12	2.57 (1.54)	7.14 (1.59)
Fear 2	M	0.00	0.00	**0.96**	0.00	0.04	2.19 (0.93)	7.24 (1.64)
	F	0.00	0.00	**0.73**	0.04	0.23	2.48 (1.17)	6.48 (1.60)
Anger 1	M	0.08	0.00	0.04	**0.88**	0.00	3.38 (2.92)	7.71 (1.55)
	F	0.27	0.00	0.04	**0.69**	0.00	4.57 (2.99)	7.86 (1.42)
Anger 2	M	0.00	0.00	0.00	**1.00**	0.00	1.95 (1.50)	7.90 (1.48)
	F	0.00	0.00	0.00	**0.96**	0.04	1.71 (1.27)	8.19 (1.29)
Anger 3	M	0.00	0.00	0.00	**1.00**	0.00	1.71 (1.31)	8.19 (1.17)
	F	0.08	0.00	0.04	**0.88**	0.00	1.90 (1.76)	7.95 (1.28)
Disgust 1	M	0.04	0.00	0.23	0.00	**0.73**	3.29 (1.71)	6.62 (1.36)
	F	0.04	0.00	0.12	0.00	**0.85**	3.52 (1.47)	5.90 (2.02)
Disgust 2	M	0.00	0.00	0.27	0.00	**0.73**	3.14 (1.74)	5.76 (1.92)
	F	0.04	0.00	0.23	0.00	**0.73**	3.19 (1.54)	5.95 (1.56)
Disgust 3	M	0.04	0.00	0.19	0.00	**0.77**	5.52 (1.78)	5.81 (1.33)
	F	0.00	0.00	0.19	0.00	**0.81**	4.55 (2.16)	5.60 (1.39)

Note: Target emotion is in bold. *SD*s included in parentheses for ratings of valence and arousal.
