# Supplementary material for: Postural Communication of Emotion: Perception of Distinct Poses of Five Discrete Emotions
Source: Front Psychol. 2017 May 16;8:710. doi: 10.3389/fpsyg.2017.00710 (PMC5432628; doi:10.3389/fpsyg.2017.00710)

Supplementary Figure 1. Images of the male and female postural poses for each discrete emotion.

Joy 1

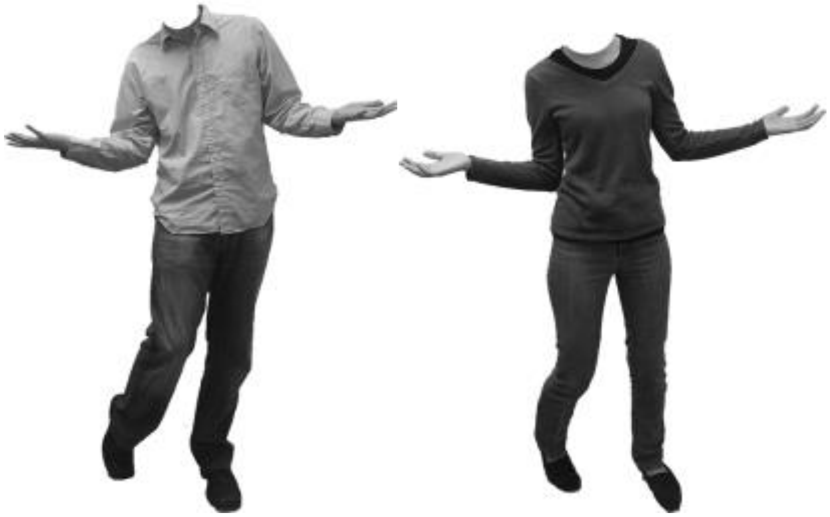

Joy 2

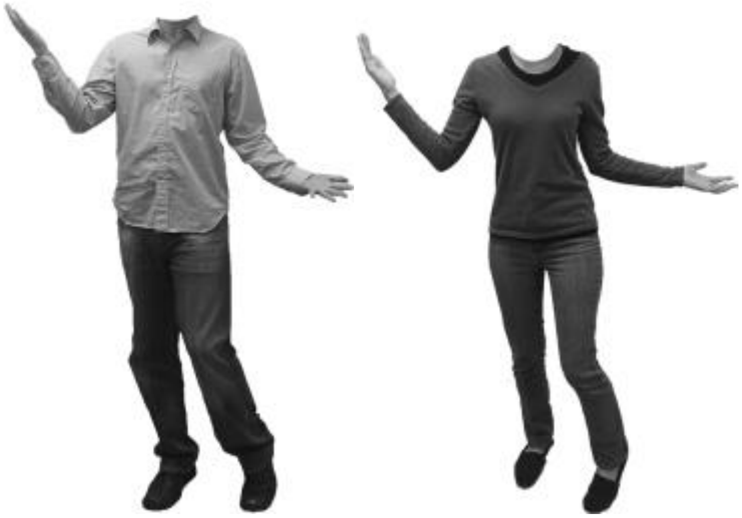

Joy 3

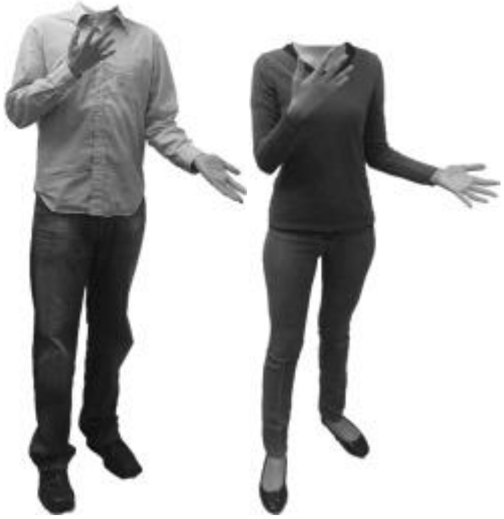

Sadness 1

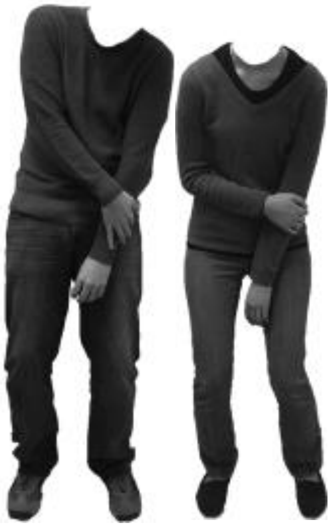

Sadness 2

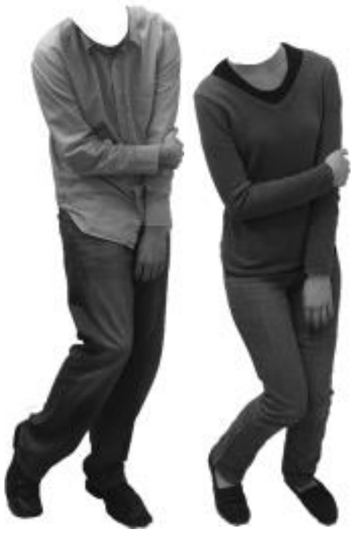

Sadness 3

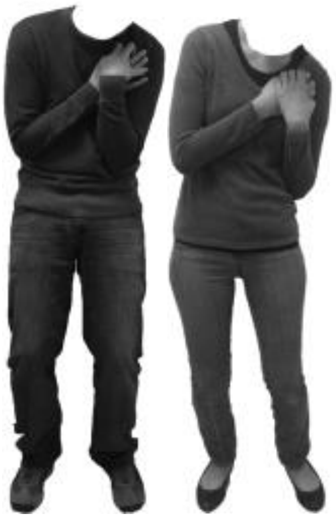

Sadness 4

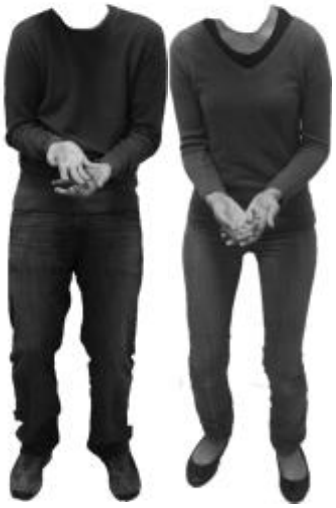

Fear 1

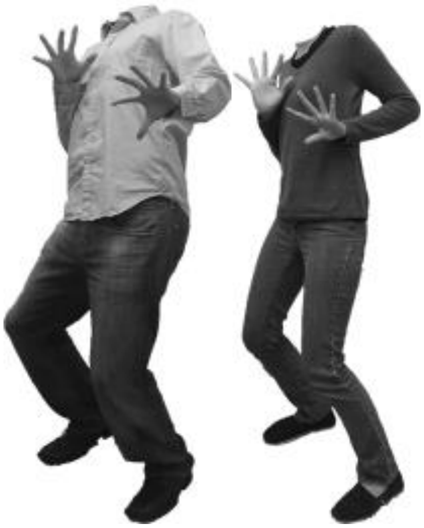

Fear 2

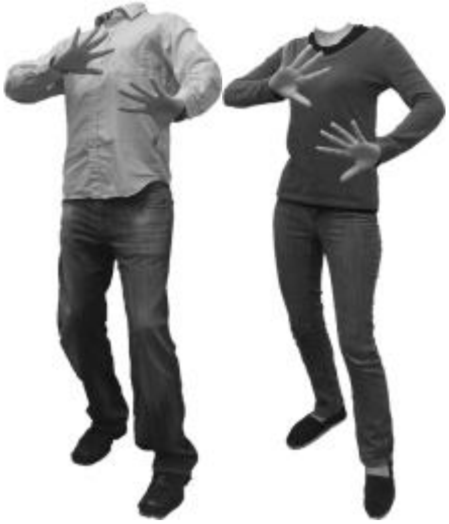

Anger 1

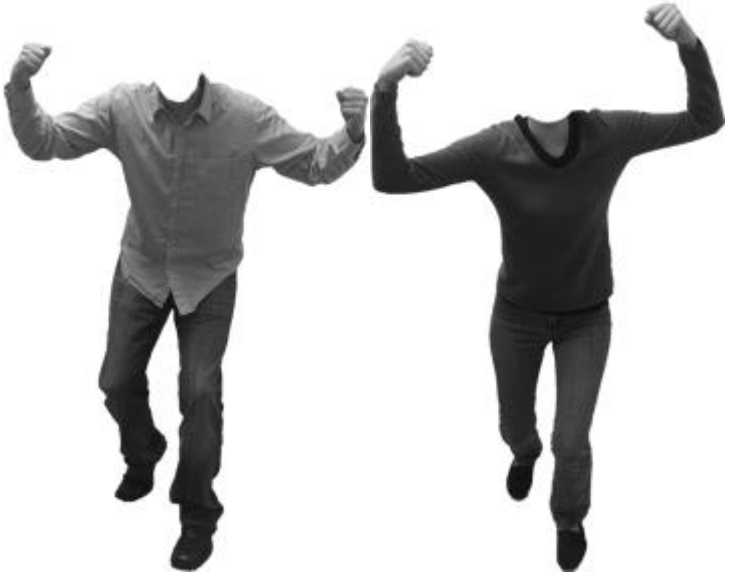

Anger 2

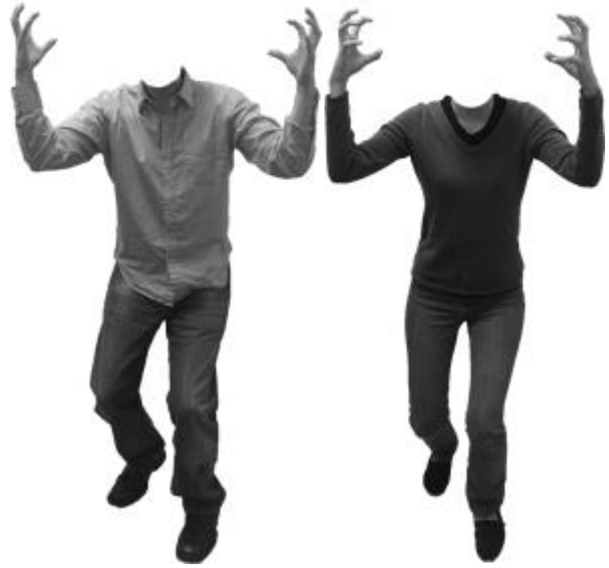

Anger 3

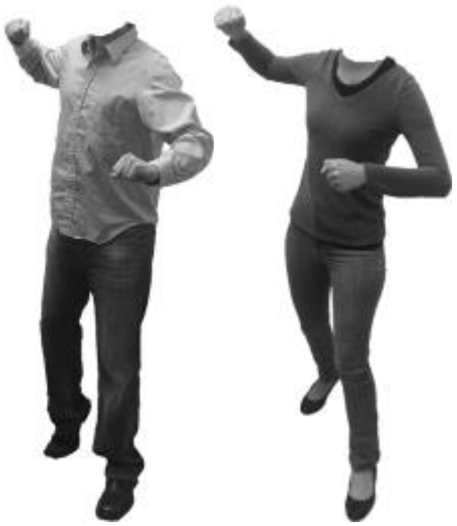

Disgust 1

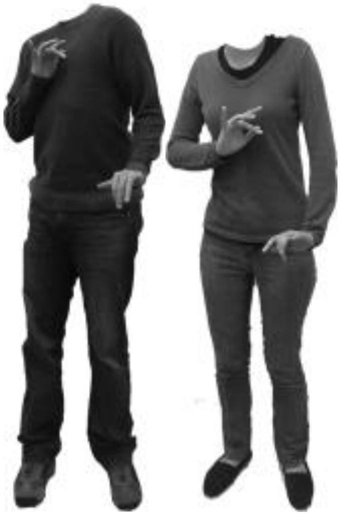

Disgust 2

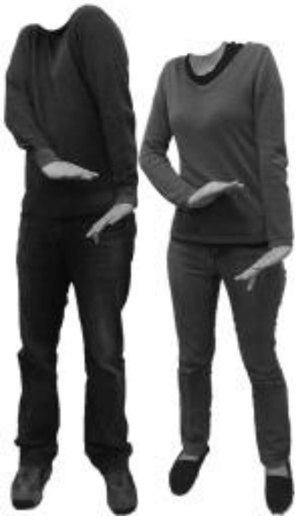

Disgust 3

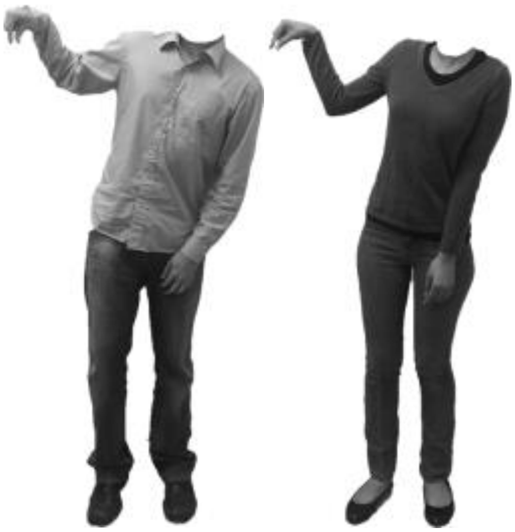

Supplement: Supplementary file 2 [file Image_1.pdf]
